# Comparison of point-of-care versus laboratory-based CD4 cell enumeration in HIV-positive pregnant women

**DOI:** 10.7448/IAS.16.1.18649

**Published:** 2013-09-16

**Authors:** Landon Myer, Kristen Daskilewicz, James McIntyre, Linda-Gail Bekker

**Affiliations:** 1 School of Public Health and Family Medicine, University of Cape Town, Cape Town, South Africa; 2 Anova Health Institute, Johannesburg, South Africa; 3 Desmond Tutu HIV Centre, Institute of Infectious Diseases and Molecular Medicine, University of Cape Town, Cape Town, South Africa

**Keywords:** point-of-care test, CD4 cell count, reliability, pregnancy, HIV, antiretroviral therapy, South Africa

## Abstract

**Introduction:**

Early initiation of antiretroviral therapy (ART) in eligible pregnant women is a key intervention for prevention of mother-to-child transmission (PMTCT) of HIV. However, in many settings in sub-Saharan Africa where ART-eligibility is determined by CD4 cell counts, limited access to laboratories presents a significant barrier to rapid ART initiation. Point-of-care (POC) CD4 cell count testing has been suggested as one approach to overcome this challenge, but there are few data on the agreement between POC CD4 cell enumeration and standard laboratory-based testing.

**Methods:**

Working in a large antenatal clinic in Cape Town, South Africa, we compared POC CD4 cell enumeration (using the Alere Pima^TM^ Analyzer) to laboratory-based flow cytometry in consecutive HIV-positive pregnant women. Bland–Altman methods were used to compare the two methods, including analyses by subgroups of participant gestational age.

**Results:**

Among the 521 women participating, the median gestational age was 23 weeks, and the median CD4 cell count according to POC and laboratory-based methods was 388 and 402 cells/µL, respectively. On average, the Pima POC test underestimated CD4 cell count relative to flow cytometry: the mean difference (laboratory test minus Pima POC) was 22.7 cells/µL (95% CI, 16.1 to 29.2), and the limits of agreement were −129.2 to 174.6 cells/µL. When analysed by gestational age categories, there was a trend towards increasing differences between laboratory and POC testing with increasing gestational age; in women more than 36 weeks’ gestation, the mean difference was 45.0 cells/µL (*p*=0.04).

**Discussion:**

These data suggest reasonable overall agreement between Pima POC CD4 testing and laboratory-based flow cytometry among HIV-positive pregnant women. The finding for decreasing agreement with increasing gestational age requires further investigation, as does the operational role of POC CD4 testing to increase access to ART within PMTCT programmes.

## Introduction

Initiation of lifelong antiretroviral therapy (ART) in eligible pregnant women is a critical intervention both to prevent the mother-to-child transmission (PMTCT) of HIV infection and to promote maternal health [1]. However, identification of ART-eligible pregnant women presents a significant barrier to PMTCT services across much of sub-Saharan Africa [2–4]. In settings where ART-eligibility is based on CD4 cell count, the limited availability of laboratory facilities for CD4 cell enumeration presents a fundamental concern [5]. Even in settings where laboratory services for CD4 testing are available, delays in the transport of specimens and return of results presents hurdles to initiation of ART in women identified as eligible [6].

Point-of-care (POC) CD4 cell count testing has been suggested as one approach to overcome the challenges related to limited laboratory access in many parts of Africa [7]. In adult HIV services, POC CD4 testing may increase the proportion of HIV-positive adults who are successfully referred to ART, with direct benefits in reducing attrition over time [8, 9]. For pregnant women, POC CD4 testing in PMTCT services may allow identification of ART eligibility at the first antenatal visit in settings where ART initiation is based on CD4 cell count in pregnancy. This may help reduce important delays to ART initiation in the context of pregnancy where rapid ART initiation is a priority for preventing HIV transmission [10].

Although POC CD4 cell count testing has the potential to enhance the identification of ART-eligible women within PMTCT services, there are few data on the test reliability of POC CD4 cell count testing in pregnancy. Previous evaluations of POC CD4 technologies have suggested reasonable agreement with laboratory-based CD4 cell enumeration (usually based on flow cytometry), with some concerns raised around operational aspects of POC tests in real-world settings [11–14]. In addition, given the haematological changes observed during pregnancy, it is plausible that the reliability of POC testing may vary by gestation age, though data are sparse [15]. We compared POC versus laboratory CD4 cell enumeration in a cross-sectional study of HIV-positive pregnant women in Cape Town, South Africa.

## Methods

The study took place in a single large antenatal clinic in Cape Town, South Africa, with a heavy burden of antenatal HIV infection (in 2012 the antenatal HIV seroprevalence was estimated to be 26%). At this clinic, all women making their first antenatal visit receive voluntary counselling and testing for HIV and undergo phlebotomy for routine antenatal screening. Venous specimens for laboratory CD4 testing were taken from HIV-positive women at this first antenatal visit. Routine CD4 cell count enumeration was conducted at the South African National Health Laboratory Services (NHLS) via Beckman-Coulter flow cytometry using panleucogated methodology, the standard of care in this setting and described in detail previously [16]. Results are returned to patients at the clinic 1–2 weeks later.

We implemented the POC Alere Pima^TM^ Analyzer (Alere Health Care, Waltham, Massachusetts, USA) into routine PMTCT services in this setting from June 2012. POC testing was conducted by either a nurse–midwife or a trained counsellor, both working in the PMTCT service, and both trained by the manufacturer. Testing used the same venous specimen that was sent for laboratory CD4 testing, with sampling using capillary tubes supplied by the manufacturer. Test procedures for the Alere Pima Analyzer followed manufacturer's guidelines, including the use of daily quality control beads and routine machine maintenance. Results are returned to patients 20–30 minutes later.

Data on women's age, prior ART use and gestation at the time of testing was abstracted from routine health care records, with approval by the Research Ethics Committee of the University of Cape Town. In analysis, we compared laboratory versus POC CD4 test results using Bland–Altman analysis for the mean difference and limits of agreement for laboratory versus POC testing [17]. Further analyses examined the test performance of the Alere Pima Analyzer where a laboratory-based test result <350 cells/µL was treated as the definition of “true” ART eligibility, with a calculation of corresponding sensitivity, specificity and likelihood ratios. Fisher's exact tests were used to compare proportions, and *t*-tests were used to compare means; all statistical tests are two-sided at *α*=0.05. Results are presented with 95% confidence intervals (CI) throughout.

## Results

A total of 546 women were tested using the POC Alere Pima Analyzer. During this period 629 test runs were conducted, with 61 women requiring multiple attempts at POC testing (four women did not receive POC CD4 testing due to repeated machine errors). The most common errors were related to inadequate or inappropriate specimen collection and/or cartridge use (e.g. air bubbles or debris in the sample; damage to cartridge during operation). Of these women, 521 women also had laboratory testing data available and are included in this analysis; there were no significant differences between those excluded and those included in the analysis. Three machines were used during this time, though the majority of tests (61%) were done with a single machine.

The median age of women participating was 27 years (IQR, 23 to 31), and 13% of women were on ART at the time of testing (Table 1). The median gestational age (among 442 women with data available) was 23 weeks (IQR, 17 to 29), and 44% and 42% of women were tested during the second and third trimesters, respectively. The median POC CD4 result was 388 cells/µL (IQR, 265 to 540; range, 6 to 1144), and the median laboratory CD4 result was 402 (IQR, 280 to 551; range, 21 to 1341). CD4 cell count values did not vary significantly with gestational age or participant demographic characteristics (not shown). Overall, 42% and 39% of women had CD4 cell counts <350 cells/µL according to POC and laboratory methods, respectively.

**Table 1:** Characteristics of HIV-positive pregnant women undergoing CD4 cell count enumeration

Pregnant women (*n=*521)	Value or *N* (%)
Median age (IQR), years	27 (23–31)
Age categories, years	
15–24	159 (31)
25–30	201 (39)
31+	150 (29)
Median gestational age (IQR), weeks^a^	23 (17–29)
Gestational age categories	
<12 weeks	55 (12)
13–18 weeks	79 (18)
19–24 weeks	117 (26)
25–30 weeks	89 (20)
31–36 weeks	74 (17)
36+ weeks	27 (6)
On ART at time of testing	66 (13)
Median laboratory CD4 cell count (cells/µL)	402 (280–551)
Laboratory CD4 result ≤350 cells/µL	203 (39)
Median Pima POC CD4 cell count (cells/µL)	388 (265–540)
Pima result ≤350 cells/µL	221 (42)

a Data on gestation at the time of CD4 cell count enumeration available on 442 of 521 participants.

**Figure 1:**
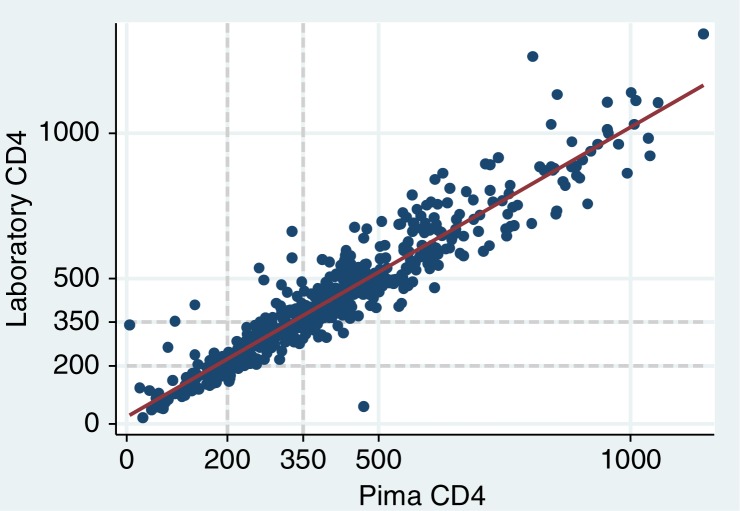
Scatterplot with best-fit line for relationship between laboratory versus Pima point-of-care CD4 cell enumeration, among HIV-positive pregnant women in Cape Town, South Africa.

**Figure 2:**
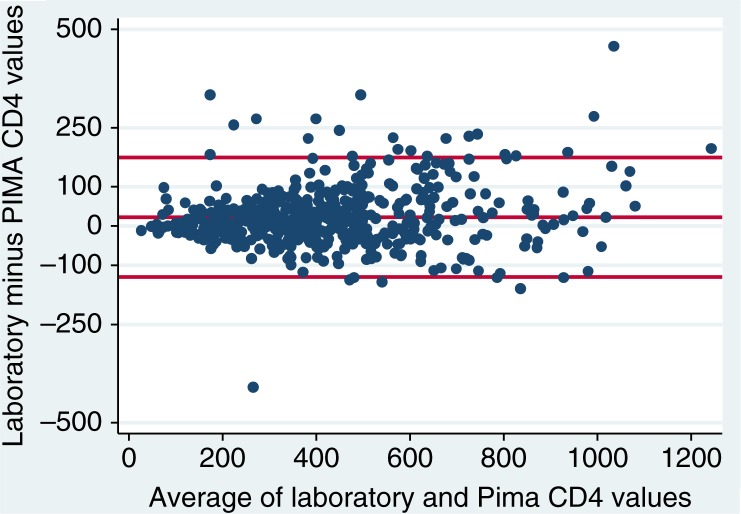
Bland–Altman plot comparing laboratory versus Pima point-of-care CD4 cell enumeration, among HIV-positive pregnant women in Cape Town, South Africa (mean difference, 22.7 cells/µL; limits of agreement, −129.2 to 174.6).

The overall correlation between the Alere Pima Analyzer and laboratory-based CD4 cell counts was high (Pearson product-moment correlation, 0.93; Spearman's rho, 0.94) (Figure 1). The Bland–Altman plot for all patients is shown in Figure 2. On average, the Pima POC test underestimated CD4 cell count relative to flow cytometry: the mean difference (laboratory test minus Pima POC) was 22.7 cells/µL (95% CI, 16.1 to 29.2), and the limits of agreement were −129.2 to 174.6 cells/µL. The Pima value was lower than the flow cytometry value for 60% of the specimens. Agreement between the enumeration modalities did not vary by the machine used or the calendar month of testing (not shown).

**Figure 3:**
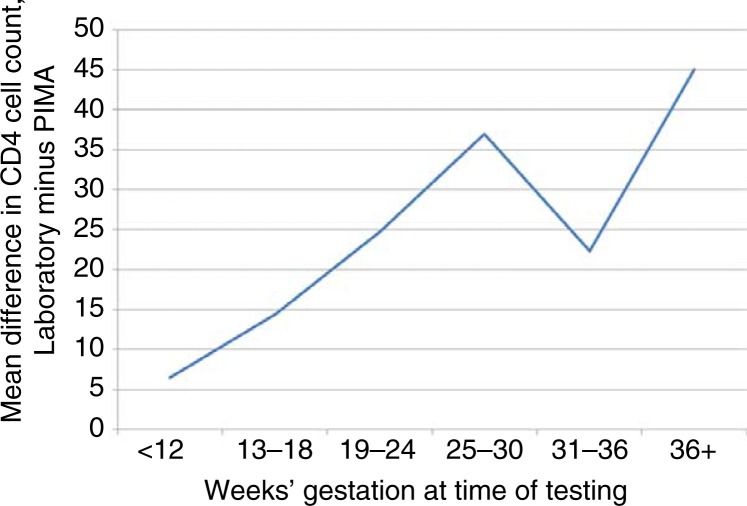
Plot of mean difference in CD4 cell count values from laboratory versus Pima point-of-care testing among HIV-positive pregnant women, by gestational age at time of testing.

Table 2 presents the agreement of tests by participant subgroups. The mean difference increased with increasing participant gestational age, from a mean difference of 6.5 cells/µL (95% CI, −16.0 to 29.0) in women tested during the first trimester to 20.6 (95% CI, 10.2 to 31.0) during the second trimester and 32.4 (95% CI, 21.4 to 43.4) during the third trimester. When further analysed by six-week categories of gestational age (Figure 3), there was a trend towards increasing differences between laboratory and POC testing with increasing gestational age; in women more than 36 weeks’ gestation, the mean difference was 45.0 cells/µL (95% CI, 11.1 to 78.7; *p*=0.04).

**Table 2:** Results of Bland–Altman analysis, overall and by participant subgroups, comparing laboratory versus Pima point-of-care CD4 cell enumeration, among HIV-positive pregnant women in Cape Town, South Africa

	Mean difference	95% CI	Limits of agreement	*p* Value for variance
All patients	22.7	16.1 to 29.2	−129.2 to 174.6	<0.001
Age categories, years				
15–24	35.1	23.7 to 46.6	−109.6 to 179.8	0.015
25–30	14.9	3.9 to 25.9	−140.9 to 170.7	0.001
31+	23.0	10.1 to 35.8	−132.6 to 178.5	0.654
Trimester at time of testing				
1st	6.5	−16.0 to 29.0	−159.9 to 172.9	0.786
2nd	20.6	10.2 to 31.0	−127.4 to 168.6	0.001
3rd	32.4	21.4 to 43.4	−121.8 to 186.6	0.005
On ART at time of testing				
Yes	21.8	5.6 to 38.0	−108.0 to 151.5	0.265
No	23.1	15.7 to 30.4	−133.2 to 179.3	<0.001

Table 3 shows the test characteristics of the Alere Pima Analyzer in detecting laboratory CD4 values ≤350 cells/µL, overall and by participant characteristics. POC CD4 testing was 92% sensitive and 89% specific in identifying women with laboratory CD4 cell counts ≤350 cells/µL; the overall likelihood ratio for the test (LR-test) was 97.6. The overall percent agreement was 90%, and the percent misclassified was 10%. In the 16 women classified as CD4≤350 cells/µL by laboratory testing, but CD4 >350 cells/µL on the Pima test, the mean difference (laboratory minus Pima) was 78 cells/µL. When the test characteristic analysis was restricted to participants with CD4 cell counts <500 cells/µL according to laboratory testing, the sensitivity remained constant (92%), but the specificity of the POC test decreased to 79% (LR-test, 43.0). The sensitivity observed in women tested during the first trimester (88%) was slightly lower than among women tested in the second or third trimesters (92%), but this difference was not statistically significant (*p*=0.47).

**Table 3:** Test characteristics of Pima point-of-care CD4 cell enumeration versus laboratory testing as gold-standard among HIV-positive pregnant women in Cape Town, South Africa. Both Pima and laboratory tests are analysed based on a threshold of 350 cells/µL

	Sensitivity	Specificity	LR+	LR−	LR test
All patients	92% (88%–95%)	89% (85%–93%)	8.6	0.09	97.6
Age categories, years					
15–24	98% (89%–99%)	89% (82%–94%)	9.2	0.02	379
25–30	91% (83%–96%)	91% (84%–96%)	10.2	0.10	103
31+	91% (81%–97%)	87% (78%–93%)	7.1	0.11	66
Trimester at time of testing					
1st	88% (68%–97%)	87% (70%–96%)	6.8	0.14	47
2nd	92% (84%–97%)	90% (83%–95%)	9.1	0.09	106
3rd	92% (83%–97%)	88% (81%–93%)	7.7	0.09	83
On ART at time of testing					
Yes	92% (79%–98%)	63% (42%–81%)	2.5	0.12	20.4
No	92% (87%–96%)	92% (88%–95%)	11.2	0.09	128

LR+: positive likelihood ratio; LR−: negative likelihood ratio; LR test: likelihood ratio for the test (diagnostic odds ratio).

## Discussion

These data suggest that in the context of pregnancy, the POC Alere Pima Analyzer appears to slightly underestimate laboratory-based flow cytometry in CD4 cell enumeration. In keeping with this, the overall sensitivity of this POC test in detecting women who are ART-eligible based on laboratory CD4 cell counts ≤350 cells/µL is high (92%). These data point to the potential role that this POC CD4 test could play in enhanced identification of ART-eligibility within PMTCT services.

This is the largest evaluation to date of POC CD4 testing in HIV-positive pregnant women, using venous blood specimens in the Alere Pima Analyzer rather than capillary blood. In smaller studies using capillary blood specimens from pregnant women, similar mean biases were documented (20.5 and 37.9 cells/µL for laboratory minus POC testing, compared to 22.7 cells/µL in these data) [11, 15]. Generally, POC CD4 testing using capillary blood specimens appears less reliable than when venous blood specimens are used, and this difference may account for some of the variability in results reported in the literature. The majority of studies comparing the Alere Pima Analyzer (using capillary or venous specimens) to laboratory-based flow cytometry have found that POC testing underestimates laboratory-based methods [11, 13, 14, 18], as documented here. In the context of POC CD4 testing to determine eligibility for ART in pregnancy, this underestimation is not a major clinical concern, particularly given the interest universal initiation of lifelong ART for all HIV-positive pregnant women [19]. However, if POC CD4 testing is to be used to monitor CD4 cell counts in HIV-positive individuals over time, this systematic bias may warrant greater attention.

Of note, the agreement in these data between the Alere Pima Analyzer and laboratory-based flow cytometry appears to decline with increasing gestational age at the time of testing, from a mean bias of 6 cells/µL in the first trimester to 45 cells/µL in women tested after 36 weeks’ gestation. The interpretation of absolute CD4 cell counts during gestation is a known concern due to haemodilution of pregnancy [20, 21], but changes in the agreement between POC tests and laboratory-based flow cytometry did not appear significant in the only previous analysis to examine this issue [15]. Increasing plasma volume in pregnancy occurs during the late first trimester and extends into the third trimester [22], and thus could account for the pattern in CD4 cell count agreement observed here; however, further investigation is required.

These data are subject to several limitations. The results come from an operational evaluation conducted at a single, large primary-care antenatal clinic, with tests using three Alere Pima Analyzer machines. While all tests were conducted using manufacturer's equipment by trained individuals, the results should still be generalized with caution. In addition, we had access only to routine patient data, so potentially important information (e.g. other haematology parameters that may help to interpret the agreement between the CD4 tests) was not available. In addition, we did not assess the assay precision (repeatability) of the Alere Pima Analyzer in this study design, and additional investigations of assay precision are warranted.

Recently, the World Health Organization recommended consideration of an approach to PMTCT based on universal ART initiation of all HIV-positive pregnant women regardless of CD4 cell count, with either cessation of ART at the end of breastfeeding for women with high CD4 cell counts (“Option B”) or continuation of ART as lifelong treatment (“Option B+”) [23]. Under these approaches, the rapid enumeration of CD4 cell counts of HIV-positive pregnant women may be less critical, as this information is not required to make short-term management decisions. However, POC CD4 testing is likely to remain an important component of PMTCT services in settings across Africa where there is limited access to laboratory-based CD4 testing, and/or countries where CD4 cell counts continue to be used to identify ART-eligible women (“Option A”), which remains the standard of care in many settings.

POC CD4 testing may play an important role in PMTCT programmes in two different settings. First, POC CD4 testing provides a practical alternative in settings where there is no regular access to laboratory-based CD4 cell enumeration. Second, in settings that have access to laboratory CD4 cell enumeration but where delays in providing CD4 results hinder PMTCT services, POC CD4 testing may provide a rapid testing strategy: by determining eligibility on the same day as HIV testing, it may be possible to expedite substantially ART initiation. These data suggest sufficient agreement between the Pima POC CD4 test and laboratory-based testing for either of these roles, but additional research is required to understand the role that POC CD4 testing can play in each of these settings. In addition, from an operational perspective, we found that 83 additional tests were required on the Alere Pima Analyzer to provide CD4 results on 546 women, leading to an excess testing proportion of approximately 15%. This error rate is consistent with a previous evaluation of the Pima CD4 test in an antenatal setting (ranging from 10 to 20%) [11]. The error rate of POC CD4 testing is not widely reported but has implications in terms of both resource requirements and patient care, and this additional “hidden” cost should be included in future economic evaluations of POC CD4 testing [24].

In summary, these data suggest reasonable overall agreement between Pima POC CD4 testing and laboratory-based flow cytometry among HIV-positive pregnant women. The preliminary findings for decreasing agreement with increasing gestational age require further investigation, as does the operational role of POC CD4 testing to increase access to ART within PMTCT programmes.
